# Multi-study analysis reveals dynamic postprandial biomarkers for metabolic syndrome by low-level fusion of multi-platform data from nutritional challenge test studies

**DOI:** 10.1186/s12263-026-00817-9

**Published:** 2026-08-17

**Authors:** Tim van den Broek, Jelle CBC de Jong, Milena Rundle, Jarlei Fiamoncini, Yoana Kiselova-Kaneva, Martien PM Caspers, Diana G Ivanova, Ben van Ommen, Jildau Bouwman, Suzan Wopereis

**Affiliations:** 1 Department of Microbiology and Systems Biology, The Netherlands Organization for Applied Scientific Research (TNO), PO BOX 2215, Leiden, 2301 CE The Netherlands; 2 Faculty of Medicine, Department of Metabolism, Digestion and Reproduction, Nutrition and Dietetic Research Group, Imperial College London, London, UK; 3 Food Research Center, Department of Food Science and Experimental Nutrition, School of Pharmaceutical Sciences, University of São Paulo, São Paulo, Brazil; 4 Department of Food and Nutrition, Technische Universität München, München, Germany; 5 Department of Biochemistry, Molecular Medicine and Nutrigenomics, Medical University of Varna, Varna, Bulgaria

**Keywords:** Meta-analysis, Metabolomics, Obesity, Diabetes, Biomarkers, Screening, Monitoring, Stratification, Precision nutrition, Postprandial response

## Abstract

**Background:**

Metabolic syndrome (MetS) is a global health issue linked to increased disease risk and economic burden. Traditional biomarkers for MetS reflect disrupted homeostasis but may fail to detect earlier stages of imbalance, i.e., an increase in allostatic load. Health can be defined as the capacity to maintain or recover homeostatic control, and this capacity can be assessed via a standardized nutritional challenge test (e.g., PhenFlex), which provokes adaptive metabolic responses. We aimed to identify a biomarker signature indicative of MetS-associated allostatic load, using data from multiple nutritional challenge test (NCT) studies.

**Results:**

A multi-study analysis was performed using data from four human studies with nutritional challenge test (*n* = 195). We identified 64 analytes with significantly altered postprandial responses in individuals with MetS (*n* = 78) compared to healthy controls (*n* = 117). Cluster analysis revealed four clusters with a distinct temporal dynamic response to the NCT. The most prominently affected analytes included total triacylglycerides, C-peptide, leptin, and a range of amino acids (e.g., L-glutamic acid, L-serine, and L-leucine). The response of a subset of these analytes to the nutritional challenge test also changed after a 12-week, 20% energy-restriction intervention in an independent trial (*n* = 72), supporting their potential as markers for monitoring intervention efficacy. Further network and ontology-based analyses revealed that the altered analytes represented key biological processes, particularly lipid and protein metabolism, as well as higher-level physiological function domains, such as liver, muscle, adipose tissue, and pancreas function. These domains were also responsive to the energy-restriction intervention.

**Conclusions:**

This study identified a robust, dynamic biomarker signature for MetS that emerges in response to a NCT. This signature reflects increased allostatic load and highlights disrupted adaptability across multiple metabolic functions. A subset of the identified markers were modifiable through dietary intervention, supporting their potential as early, actionable biomarkers of metabolic health.

**Supplementary Information:**

The online version contains supplementary material available at https://doi.org/10.1186/s12263-026-00817-9.

## Background

Metabolic syndrome (MetS) is a widespread and rapidly growing global health issue [1, 2], accompanied by a significant economic burden [3]. A key step in reducing this burden is the identification of biomarkers associated with MetS. These biomarkers could be valuable for early risk screening, monitoring disease progression, and evaluating treatment efficacy. Current biomarkers primarily reflect disruptions in homeostatic systems (e.g., dyslipidemia or microalbuminuria) [4]. However, it remains unclear whether these biomarkers can reliably signal early imbalances in health, i.e., an increase in allostatic load, before homeostasis is compromised. For the discovery of biomarkers that are indicative of allostatic load, a new perspective on health is needed.

Physiological health can be regarded as the ability to adapt to and cope with day-to-day challenges such as strenuous exercise or a dietary challenge without drifting into dysregulation [5]. This perspective on health has inspired several of our previous studies [6–10]. These studies have used nutritional challenge tests (NCT) and were designed to quantify phenotypic flexibility as a new way of measuring health [11]. Phenotypic flexibility associated with an adaptive homeostatic response can best be measured during a period of homeostatic disturbance [12]. The postprandial response is of particular interest to measure health since it reflects how well the body handles real-world metabolic challenges, a key predictor of disease [13]. Therefore, these studies used a standardized high fat NCT to evoke an adaptive response, which was consequently quantified by performing metabolomics on collected blood samples.

In order to increase power and allow for a more sensitive analysis, we performed a multistudy analysis using metabolomics data of four studies. The included studies were originally performed to serve different goals, however, the employed methodologies were similar and baseline data can be merged. By combining these datasets, we set out to find a biomarker signature for MetS which indicates an increase in allostatic load. Additional analyses were performed to gain insight into which biological processes and physiological function domains are associated with the increased allostatic load resulting from MetS. Lastly, we evaluated the responsiveness of the identified biomarker signature to a dietary intervention using an independent weight-loss study (NutriTech) [14], thereby providing an external validation of the biological relevance and modifiability of the identified markers. We hypothesize that MetS is characterized by a reproducible dysregulation of the postprandial metabolic response, reflecting increased allostatic load and impaired phenotypic flexibility, and that part of this signature is modifiable through an energy-restriction intervention. This novel approach, metabolically challenging persons living with MetS and integrating data across multiple studies, provides a robust framework for identifying biomarkers and underlying mechanisms of MetS.

## Methods

### Study selection

Data were obtained from the Phenotype database (https://dashin.eu/interventionstudies/). This is an open source application built to store and retrieve biological study data. Study selection was performed on the 14th of April 2025 on the basis of three criteria: studies were included if (1) both healthy control and persons living with MetS were recruited, (2) a NCT was performed and (3) multiplatform analysis was performed including metabolomics on blood samples collected during the NCT. We only used baseline data from selected studies, prior to any intervention. Four studies with a total of 195 participants were included on the basis of their compatibility as judged by the study demographics and overlap in measurement platforms (see Table 1) [8, 9, 15, 16]. These four studies were used to create the network that served as the basis of the multi-study analysis. All four included studies were conducted in accordance with the Declaration of Helsinki. The study protocols of two studies were approved by the independent Medical Ethics Committee METOPP located in Tilburg, the Netherlands [15, 16], the study protocol of one study was approved by the independent Medical Ethics Committee of the Leiden University Medical Centre [9] and the study protocol of one study was approved by the independent Medical Ethics Committee of the Foundation ‘Evaluation of Ethics in Biomedical Research’ located in Assen, the Netherlands [8]. All participants provided an informed consent for inclusion before they participated in the studies.

The four included studies were used for biomarker discovery. Subsequently, data from the independent NutriTech study [14] (also obtained from https://dashin.eu/interventionstudies/) were included to evaluate whether the identified biomarkers were responsive to a dietary intervention and could therefore serve as actionable markers of metabolic health [14]. Briefly, seventy-two individuals with overweight or obesity participated in a 12-week dietary intervention. They were randomly assigned to either a weight loss group with a 20% energy-restricted diet with a dietary profile aligned to Dietary Approaches to Stop Hypertension (DASH) or a weight maintenance group with a dietary profile that was based on the average intake in the EU. Metabolic markers were measured through plasma metabolomics during a NCT before and after the intervention. More details of the study design, metabolomics methodology and analysis are given elsewhere [14].

### Nutritional challenge tests

All included studies used high fat challenge tests as NCT, although the composition and sampling protocols differed between studies. Three of the five studies, namely van den Broek et al. [8], Wopereis et al. [9] and Rundle et al. [14], used the standardized PhenFlex challenge test. The test was administered in the morning after an overnight fast of at least 10 h. The challenge consisted of a 400 mL beverage providing 3950 kJ (950 kcal), containing 60 g fat, 75 g glucose, 5 g polysaccharides, and 20 g protein. Venous blood samples were collected before consumption of the beverage (t = 0) and at 0.5, 1, 2, 4, 6, and 8 h thereafter. Participants were not permitted to consume food or drinks during the test period, except for water [17].

In the study of Bakker et al. [15] the participants consumed a fat-load test after a 4-hour fast following a standardized light breakfast. The challenge consisted of a 500-mL milkshake containing high-fat dairy products, providing 46.1 g fat and a total energy content of 2945 kJ. Venous blood samples were collected at baseline (t = 0) and at 0.5, 1, 2, 3, 4 and 6 h after consumption.

In the study of Kardinaal et al. [16], participants consumed a 500-mL high-fat challenge consisting of 53% whipping cream, 3% sugar, and 44% water. The challenge provided approximately 3200 kJ and contained 80 g fat, 8 g protein and 16 g carbohydrates. Following an overnight fast, blood samples were collected before consumption (t = 0) and 1, 2, 4, 6 and 8 h after consumption.

### Subgrouping of participants

Participants from the included studies were divided into either healthy control or a MetS group by an automated procedure based on the WHO criteria for MetS. The World Health Organization 1999 criteria for MetS require the presence of at least one of: Type 2 diabetes mellitus, impaired glucose tolerance, impaired fasting glucose or insulin resistance, AND two of the following [18]:

- Blood pressure: ≥ 140/90 mmHg.
- Dyslipidemia: triacylglycerides (TGs): ≥ 1.695 mmol/L and high-density lipoprotein cholesterol (HDL-C) ≤ 0.9 mmol/L (male), ≤ 1.0 mmol/L (female).
- Central obesity: waist: hip ratio > 0.90 (male); > 0.85 (female), or body mass index > 30 kg/m2.
- Microalbuminuria: urinary albumin excretion ratio ≥ 20 µg/min or albumin to creatinine ratio ≥ 30 mg/g.

This resulted in the inclusion of 117 healthy control and 78 persons living with MetS, see Table 1.

**Table 1:** An overview of participants per included study and in total

Study	Number of healthy persons	Number of persons with MetS	Total
Kardinaal et al. [16]	10	9	19
Bakker et al. [15]	20	16	36
Van den Broek et al. [8]	62	38	100
Wopereis et al. [9]	25	15	40
Total (n)	117	78	195

### Cross-study integration

Study datasets were imported from the Phenotype database using the processed measurement values from each study. Study-specific sampling labels and relative sampling times were recoded to consistent names. Analytes were matched across studies using the feature-mapping table provided with the database export. ChEBI identifiers were used for analytes and UniProtKB identifiers for proteins when these identifiers were available. Features without a common identifier retained their original study-specific name.

Group comparisons were performed separately within each study. Differences in area under the curve between the healthy control and metabolic syndrome groups were assessed using Student’s t-tests, and differences in time-resolved response profiles were assessed using ANOVA. The resulting study-level p-values for each matched feature were combined using Fisher’s method and corrected for multiple testing using the Benjamini-Hochberg procedure.

Standardized mean differences (expressed as Cohen’s D) were calculated for each feature, study, and sampling time point. The study-level effect estimates were combined using inverse-variance weighting, so that estimates with greater precision contributed more strongly to the pooled result.

Fisher’s method and inverse-variance pooling of Hedges’ (g) were used as complementary meta-analytic approaches. Fisher’s method combined the evidence from study-specific statistical tests to complement the pooled Hedges’ (g) summaries of the magnitude and direction of group differences.

#### t-test and ANOVA aggregation

All analyses were performed using R Statistical Software (v3.3.0; R Core Team 2016). Two classical methods were used to evaluate features of the four individual studies. Group differences within studies were tested using a Student’s t-test on the area under the curve (AUC) for each of the analyzed features, while ANOVA was used on the time-resolved data for each feature. The calculations generating the AUC values used in the analysis were based on incremental areas under or over the baseline, using the first measurement (t = 0) as reference. In this paper, the term area under the curve (AUC) refers to values both under and above the reference; these were delineated as negative AUC (AUC-) and positive AUC (AUC+). In order to determine which features were significantly different between groups not only within a study but also over the included studies, an aggregated P-value was calculated by collating each of the individual statistical test performed within the studies. The aggregation of p-values was performed according to Fisher’s method [19]. Combined p-values are calculated according to where *p*_i_ is the p-value for the *i*^th^ hypothesis test.

$$\:{X}_{2k}^{2}\sim-2\sum_{i=1}^{k}\mathrm{log}\left({p}_{i}\right)$$

The resulting aggregated p-values were then corrected for false discovery rates using the Benjamini-Hochberg procedure [20].

#### Effect size calculation

The effect size (*d*) is the standardized mean difference between two groups [21, 22].

$$\:d=\frac{{\stackrel{-}{x}}_{1}-{\stackrel{-}{x}}_{2}}{{S}_{p}}$$

Here, $\overline{X}_{1}$ and $\overline{X}_2$ are the group means and $S_{p}$ is the pooled standard deviation and computed as:

$$\:{S}_{p}=\sqrt{\frac{\left({n}_{1}-1\right){S}_{1}^{2}+\left({n}_{2}-1\right){S}_{2}^{2}}{{n}_{1}+{n}_{2}-2}}$$

where$\:{n}_{1}$ and $\:{n}_{2}$, and $\:{S}_{1}^{2}$ and $\:{S}_{2}^{2}$ are the sample sizes and variances of both groups, respectively. The estimate of *d* is biased with small sample sizes. This is corrected for in Hedges’ effect size measure *g* which includes a multiplicative correction factor and is defined as [23]:

$$\:g=\left(1-\frac{3}{4({n}_{1}+{n}_{2)}-9}\right)\cdot\:d$$

The standard error (*SE*) of Hedges *g* is calculated as:

$$\begin{gathered} \:S{E_g} \hfill \\ = \left( {1 - \frac{3}{{4({n_1} + {n_{2)}} - 9}}} \right) \cdot \:\sqrt {\frac{1}{{{n_1}}} + \frac{1}{{{n_2}}} + \frac{{{d^2}}}{{2({n_1} + {n_{2)}}}}} \hfill \\ \end{gathered}$$

For all measured outcomes, *g* and its variance were calculated for every available study and on every available time point. Next, the corresponding effect sizes from the various studies (i.e., all the effect sizes for a given outcome and time point) were averaged using the inverse of their variance (*IV*) as the weighing factor [24]. Therefore,

$$\:{g}_{overall}=\sum_{i=1}^{N}\raisebox{1ex}{$\left({g}_{i}\cdot\:\frac{1}{{SE}_{{g}_{i}}}\right)$}\!\left/\:\!\raisebox{-1ex}{$\sum_{i=1}^{N}\frac{1}{{SE}_{{g}_{i}}}$}\right.$$

where N is the total number of studies running from i to N. The standard error around $g_{overall}$ is simply the squared sum of the inverse variance weights and hence calculated as:

$$\:{SE}_{{g}_{overall}}=\sqrt{\sum_{i=1}^{N}{\left(\frac{1}{{SE}_{{g}_{i}}}\right)}^{2}}$$

The 95% confidence interval is equal to the point estimate for the effect size plus and minus 1.96 times its corresponding standard error. These steps result in an estimate of $g_{overall}$ for every outcome and every time-point that at least two studies have in common. Hedges’ *g* is a relative measure, but convention defines <|0.2| as a small effect, >|0.2| and <|0.5| as a moderate effect and >|0.5| as a large effect [25]. When the lower limit of the 95% confidence interval was ≥ |0.2| we concluded that the overall effect size was relevant and meaningful.    

### Study design and main outcomes of independent NutriTech study

Data from the independent NutriTech study [14] were used to evaluate if the identified biomarkers were responsive to a dietary intervention. In short, the Nutritech study was a randomized controlled dietary intervention study in 72 overweight and obese adults. Participants were assigned to a 12-week energy-restriction (− 20% energy intake) or weight-maintenance diet. Nutritional challenge tests (PhenFlex challenge) were performed at baseline and after intervention, with extensive metabolic phenotyping including clinical chemistry (Supplementary Table 1). The weight loss group achieved a mean weight reduction of 5.6 kg and showed improvements in body composition, accompanied by changes in postprandial metabolic responses [14].

### Network creation

Results from the effect size estimation and the aggregated statistical analyses were combined and presented in graphical form as a network. The nodes in this network represent features which were selected by criteria pertaining to their statistical power. Three types of analyses results were included. The nodes in this network represent features selected according to predefined statistical criteria. A feature was included if it had a p-value below 0.05 in either the aggregated ANOVA or t-test analysis or an absolute estimated effect size greater than 0.2 at two or more time points, which could be consecutive or non-consecutive. This method resulted in a list of 64 analytes originating from all methods of analysis.

The network was constructed by using the mutual k-nearest neighbors (m-knn) graph of the features from the aforementioned feature list. In a m-knn graph two nodes are connected if the two nodes are among each other’s k most similar nodes with an edge weight equal to their similarity. This graph was constructed using the cosine similarity of the included analytes. Cosine similarity provides a measure of similarity equivalent to Pearson’s correlation on mean centered data. Similarity was calculated using all data across all included studies for each biomarker. When two analytes did not have equivalent amounts of data (e.g., one was measured more often or in case of missing data), only pairwise complete observations were used to calculate similarity. The resulting similarity matrix was used to construct the mutual k-nearest-neighbor graph using (k = 8). This value was selected using the previously applied heuristic (2ln(n)), where (n) represents the number of nodes [26]. For the 64 analytes included in the network, (2ln(64) = 8.32), which was rounded to eight neighbors.

For each node, the fraction of studies in which the analyte was found to be significant between groups was added as a property. Furthermore, every analyte that was found to be significantly different in concentrations between groups in the NutriTech study in fasting or during the NCT was annotated as such. The weight of the edges within the network was determined by the similarity value of the analyte that it connects.

All analytes included in the network are associated with certain biological processes. Each of these biological processes are in turn related to their respective physiological function domains. This ontological information was collected in an earlier effort and published by several of the current authors [12]. This information was utilized in the creation of the biological process and tissue domain level network visualization. Here, the ontological information associated with each node in the molecular-level network was used to condense the molecular nodes to produce a process level network. In this ontology every analyte is associated with a certain biological process. Nodes within one process were combined into a new node that represented this respective process. The size of this node is determined by the number of analytes within the process. Edges that connect these nodes were weighted by the sum of the edges that connect the analytes that underlie these functions. In turn, the process level network was condensed to a domain level network. Nodes were again grouped into new nodes signifying domains as represented by the analytes. The edges were again weighted by the sum of the edges that connect the analytes that underlie the domains. These networks were visualized using Gephi version 0.9.1 [27].

### Network analysis

The community detection algorithm used for the clustering of the nodes is an implementation of the modularity algorithm published by Blondel et al. [28]. This algorithm determines the presence of communities in the network by looking for areas within the network that contain nodes which are more densely connected together than to the rest of the network. The implementation of this algorithm used in this paper includes a tunable resolution parameter as published by Lambiotte et al. [29], which allows to parameterize the sensitivity of the community detection. Lower sensitivity allows for more disconnectedness within communities. A resolution of 2 was selected pragmatically to avoid excessive subdivision of the relatively small 64-node network and to obtain a limited number of communities representing broad, biologically interpretable patterns of response to the NCT. The resolution value was not formally optimized. This means that the identified communities were more distinct and thus have less overlap between communities in their type of response to the NCT.

## Results

### Subgrouping of participants

An overview of baseline participant characteristics that were subdivided into healthy control or MetS from a total of four included studies is given in Table 2. Sex was equally distributed among the two groups (24% and 28% females in the healthy control and MetS group, respectively). All other variables were significantly higher in the MetS group compared to the healthy control, except for the ratio HDL/LDL, which was significantly lower in the MetS group as compared to the healthy control group.

**Table 2:** Data are expressed as mean ± SD

Variable	Healthy control group (*n* = 117)	Metabolic syndrome group (*n* = 78)	False discovery rate *p*-value
Sex (%Female)	24%	28%	
Age (yrs)	41 ± 12	53 ± 11	**< 0.001**
BMI (kg/m^2^)	23.6 ± 3.9	29.5 ± 4.0	**< 0.001**
Body fat (%)	17.4 ± 7.4	31.5 ± 9.2	**< 0.001**
Glucose (mmol/L)	5.6 ± 1.2	6.6 ± 2.2	**< 0.001**
Insulin (ng/mL)	0.3 ± 0.3	0.5 ± 0.3	**< 0.001**
Cholesterol (mmol/L)	4.2 ± 1.4	4.9 ± 1.6	**< 0.001**
HDL (mmol/L)	1.4 ± 0.3	1.2 ± 0.3	**< 0.01**
LDL (mmol/L)	2.8 ± 1.1	3.4 ± 1.0	**< 0.001**
Ratio HDL/LDL	0.6 ± 0.3	0.4 ± 0.1	**< 0.001**
Triglycerides (mmol/L)	1.2 ± 0.7	1.8 ± 0.9	**< 0.001**
Systolic blood pressure (mmHg)	124 ± 10.2	135 ± 14.2	**< 0.001**
Diastolic blood pressure (mmHg)	75 ± 8.7	81.6 ± 10.8	**< 0.001**

### Cluster analysis reveals distinct temporal dynamics of clusters of analytes in response to a nutritional challenge test

**Fig. 1:**
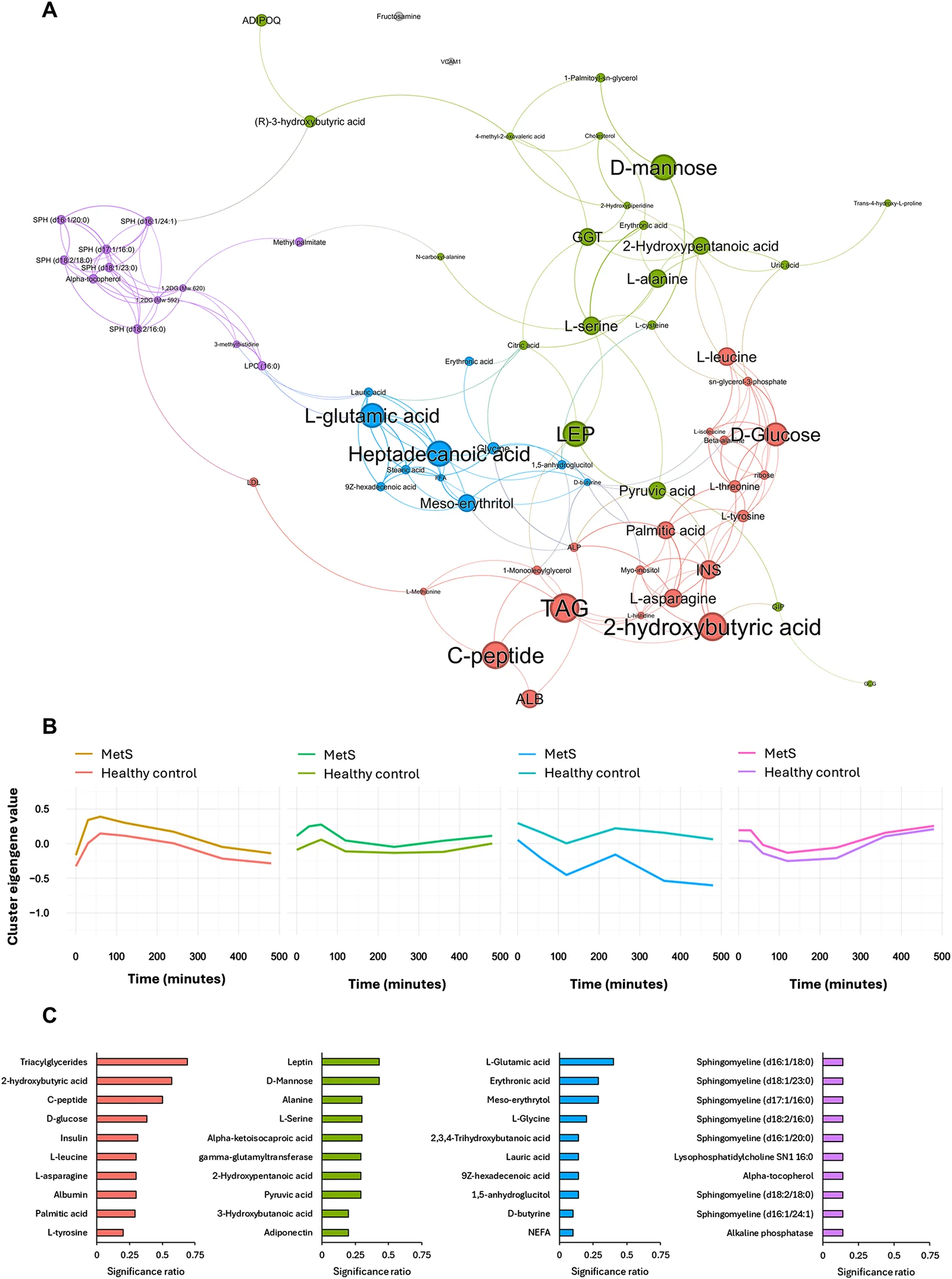
Cluster analysis of the molecular signature of metabolic syndrome. (**A**) Nodes represent analytes within the signature of metabolic syndrome. Edge thickness indicates the similarity between linked nodes. Node size indicates the significance ratio (total number of significant outcomes compared to total possible amount of significance). The color of the nodes indicates their cluster membership as determined by a community detection algorithm. (**B**) Panels show the NCT response profiles to the nutritional challenge test for each of the four identified clusters (corresponding to their color). The graphs shown are normalized and their values cannot be compared between the graphs. They serve to show the approximate difference in prototypical response types between the clusters as well as the approximate difference between the metabolic syndrome and healthy groups for the prototypical response within a cluster. (**C**) Per cluster the top 10 analytes are presented ranked on their significance ratio

The multi-study analysis identified 64 analytes that showed different dynamic response profiles in healthy control and persons living with MetS during the NCT (Fig. 1A). See Supplementary Table 2 for a full overview of the analytes. These analytes were used as input for network analysis and four different clusters were identified that each showed a distinct temporal response type to the NCT (Figs. 1B-C). The red cluster contains analytes (e.g., TAGs, 2-hydroxybutyric acid and C-peptide) which generally initially increased in concentration until t=60 min and then started to transiently decline in concentrations. The green cluster contains analytes (e.g., leptin, D-mannose and L-alanine) which also initially increased in concentration until t=60 min and then rapidly declined in concentrations and remained stable from t=120 min onwards. Analytes in the blue cluster (e.g., L-glutamic acid, erythronic acid and meso-erythritol) initially decreased in concentrations until t=120 min and thereafter increased in concentration again until t=240 min. In the blue line specifically, a further drop in concentration can be observed from t=240 min onwards. In the purple cluster, the analytes (e.g., sphingomyelines (d16: 1/18:0), (d18:1/23:0) and (d17:1/16:0)) initially decreased in concentration but started to increase again from t=120 min onwards. These results indicate the presence of distinct clusters of analytes based on the temporal dynamics of their response to the NCT.

**Fig. 2:**
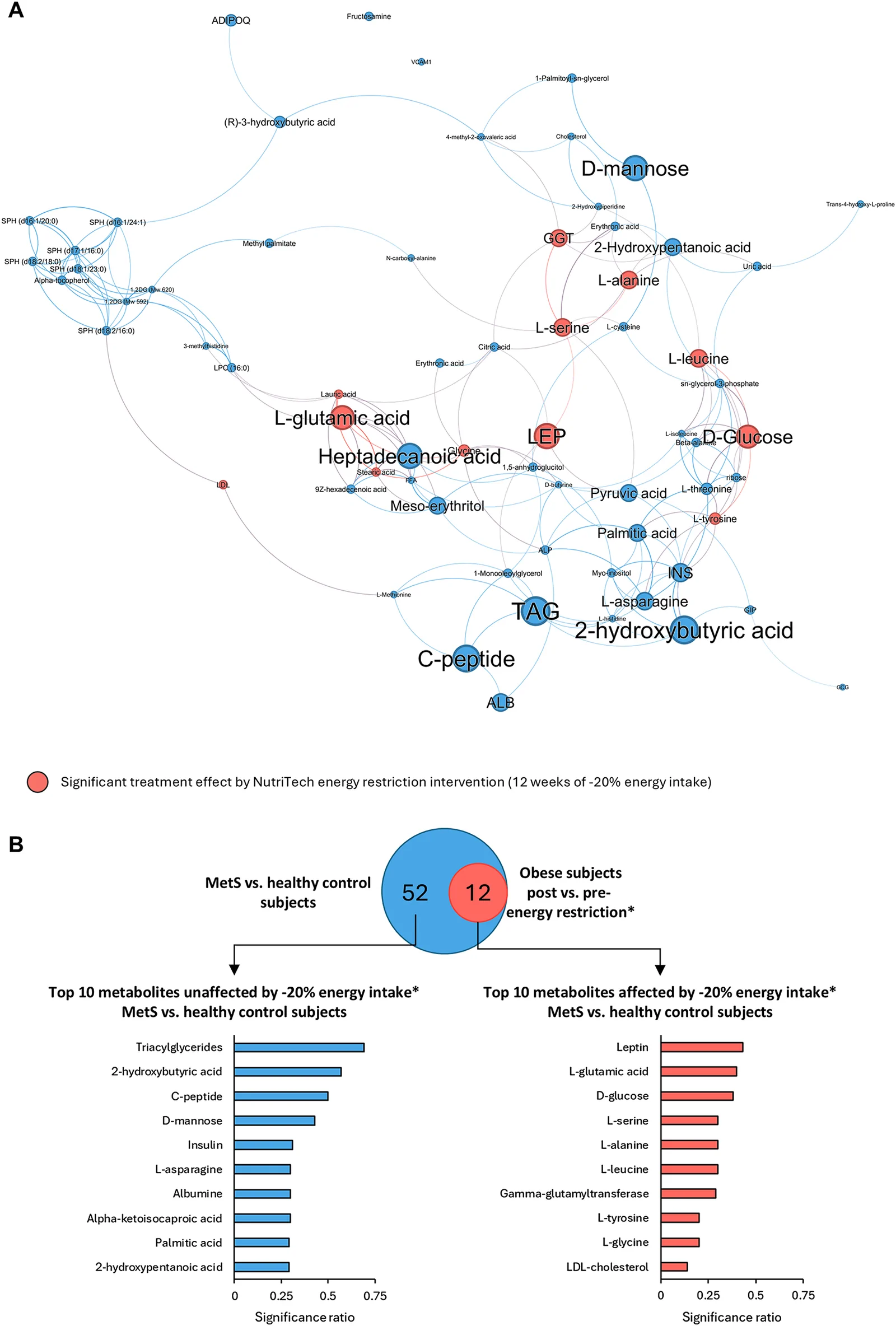
The effect of the NutriTech energy-restriction intervention (*12 weeks of -20% energy intake in healthy obese subjects) on the molecular signature of metabolic syndrome. (**A**) The orange color indicates that a significant treatment effect of the NutriTech energy-restriction intervention was found on the respective analyte. Nodes close together have higher similarity than remoter nodes as a result of the force-directed node layout algorithm. (**B**) The Venn diagram displays the number of analytes regulated by MetS alone or also by the NutriTech energy-restriction intervention. Lower panels display the top 10 analytes regulated by MetS alone or also by the NutriTech energy-restriction intervention. Significance ratios are based on the multi-study comparison of MetS vs. healthy control subjects

Out of the 64 identified analytes (as displayed in Fig. 1), 12 were affected as well by a 12-week trial of 20% energy-restriction and included leptin, L-glutamic acid, glucose, L-serine, L-alanine, L-leucine, gamma-glutamyltransferase, L-tyrosine, glycine and LDL-cholesterol (Figs. 2A-B).

### Lipid and protein metabolism are strongly represented by the MetS signature and affected by energy-restriction

**Fig. 3:**
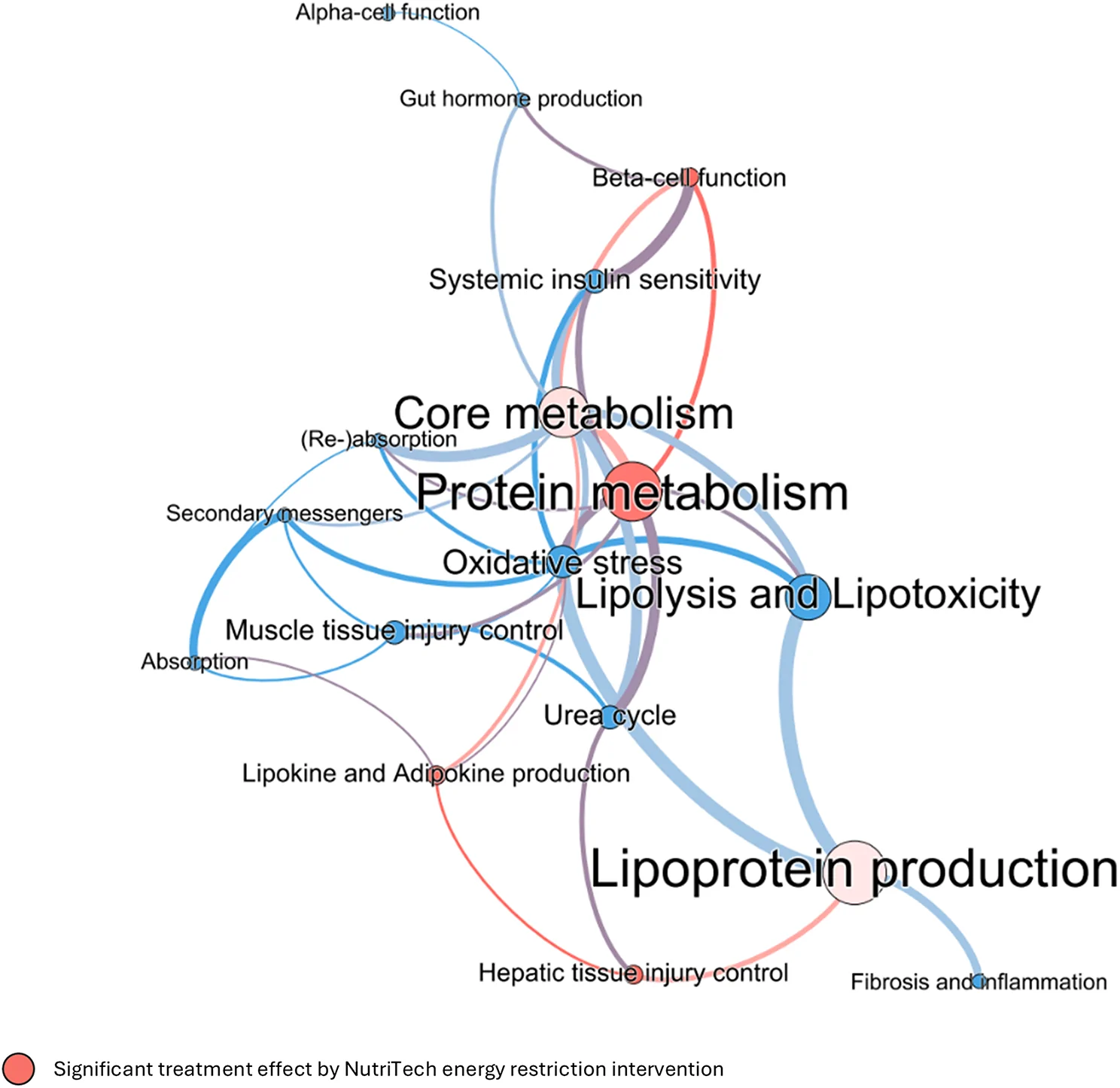
The biological process level representation of the metabolic syndrome signature network. The size of the nodes indicate the quantity of analytes condensed into each biological process that were present in the signature. The intensity of the orange hue indicates the fraction of analytes in the respective process that show a treatment effect by the NutriTech energy-restriction intervention response. Edge thickness indicates the number of shared analytes between the processes on a molecular level (as displayed in Figs. 1 and 2)

The analytes with significantly different NCT response profiles (as displayed in Figs. 1 and 2) were mapped to discrete biological processes. The largest biological processes presented by the MetS analyte dynamic signature were related to protein metabolism, lipoprotein production, lipolysis and lipotoxicity and core metabolism. Of those, protein metabolism is mainly affected by energy-restriction and to a lesser extent core metabolism and lipoprotein production (Fig. 3). Smaller biological processes represented by the metabolic signature were oxidative stress, beta-cell function, gut hormone production, alpha cell function, systemic insulin sensitivity, muscle tissue injury control, hepatic injury control, urea cycle, lipokine and adipokine production, secondary messengers, absorption and re-absorption, fibrosis and inflammation. Of these processes, which included only a few quantified analytes, beta-cell function, lipokine and adipokine production, and hepatic tissue control were significantly affected by the energy-restriction (Fig. 3).

### Liver, muscle, adipose tissue and pancreas health are strongly represented by the MetS signature and affected by energy-restriction

On a higher level of abstraction, these biological processes were grouped into tissue domains. The following domains were represented by the network, in order of size; liver health, muscle health, adipose tissue health, systemic stress response, kidney health, pancreas health, gut health and inflammation. Liver, muscle, adipose tissue and pancreas health were all affected by the energy-restriction.

**Fig. 4:**
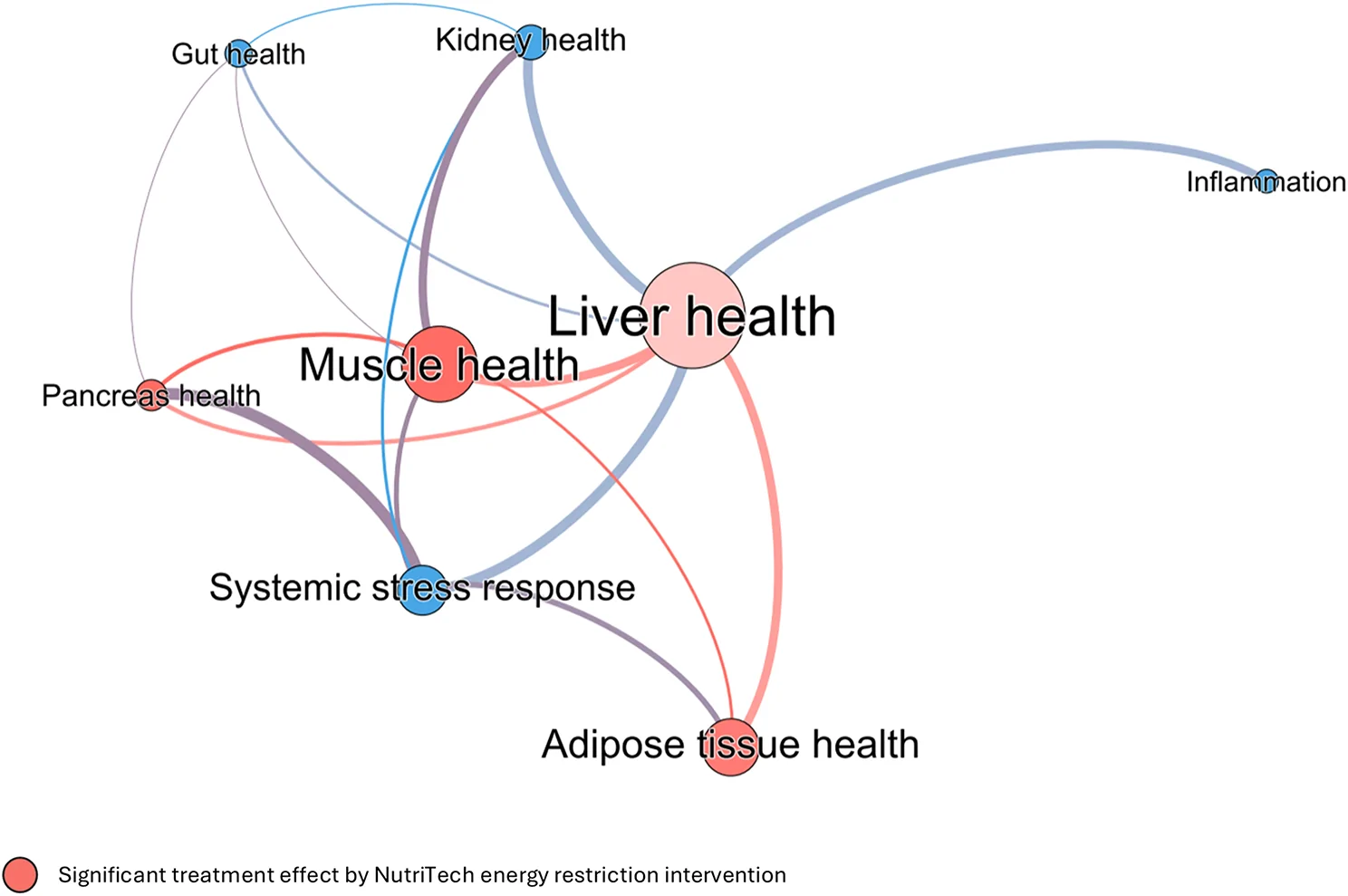
The physiological function domain level representation of the metabolic syndrome signature network. Processes (as displayed in Fig. 3) are condensed into their respective domains. Size of the nodes indicate the quantity of processes representing each domain. The intensity of the orange hue indicates the fraction of processes in the respective process that show a treatment effect by the NutriTech energy-restriction intervention response. Edge thickness indicates the number of shared edges between the domains on a process level (as displayed in Fig. 3)

## Discussion

The aim of this study was to identify a biomarker signature for MetS to indicate an increase in allostatic load. Biomarkers were identified by a dysregulated response to a mixed-meal NCT, rather than by assessing concentrations of analytes in samples collected during overnight fasting conditions. This innovative methodology stems from a novel perspective on health, which can be defined as the capacity to maintain or recover homeostatic control [30]. By combining data from four different studies, we provided a robust insight into the analytes (Figs. 1 and 2), biological processes (Fig. 3) and physiological function domains (Fig. 4) affected by MetS. To perform this multi-study analysis, we developed a new method to aggregate data from multiple studies, performed data analysis and visualized these outcomes into one multifaceted outcome. Thereby, we identified 64 analytes that showed an altered response to a NCT in the presence of MetS. In addition, we identified a subset of analytes that were modifiable through dietary intervention, supporting their potential as early, actionable biomarkers of metabolic health. For many of the reported parameters, this paper provides the first evidence that MetS alters their response to a NCT.

This overview of MetS-related analytes included both commonly and less commonly reported markers associated with metabolic disturbances (Fig. 1) [31]. TAGs (triacylglycerols), with a fasting threshold of ≥ 1.695 mmol/L, are among the diagnostic criteria for MetS [18]. Their inclusion in this metabolic signature underscores not only their role in baseline assessment, but also their dynamic response to NCTs, as widely reported [12, 16]. A similar argument applies to glucose, another key diagnostic marker, whether as impaired fasting glucose or postprandial glucose dysregulation. Although insulin is not (yet) part of the standard diagnostic criteria for MetS [18], altered insulin responses are closely tied to insulin resistance, which is central to the pathophysiology of MetS. Interestingly, C-peptide had a higher significance ratio than insulin, suggesting that an aberrant response of C-peptide was more pronounced than the impaired response of insulin in the presence of MetS (Fig. 1). However, since C-peptide and insulin have a firm mechanistic connection, both of their occurrences are likely to represent the same abnormality in metabolic health [32]. Leptin is another analyte with a higher significance ratio within the signature that has been widely associated with metabolic disturbance. Most research around leptin appears to focus on the fasting levels of leptin that relate to varying degrees of metabolic disruption. In line with the current findings, the leptin response to a mixed-meal tolerance test has been reported to be affected by obesity [33] and type 2 diabetes mellitus [34]. The current results demonstrate that the postprandial leptin response is among the parameters with the highest significance ratio differentiating between healthy and MetS individuals.

L-glutamic acid, L-serine, L-leucine and L-alanine were (in order of significance) the most discriminatory amino acids in the signature (based on significance ratio). Other amino acids showing a smaller effect were L-glycine, L-isoleucine, L-tyrosine, L-histidine, L-cysteine, L-asparagine, L-threonine and L-methionine. The presence of this wide range of amino acids suggests a broadly dysregulated amino acid metabolism in the presence of MetS. This is consistent with prior literature, particularly highlighting alterations in branched-chain and aromatic amino acids, which have been repeatedly associated with insulin resistance and metabolic disease [35–37]. While most existing studies focus on fasting amino acid levels, our findings emphasize differences in postprandial responses between individuals with MetS and healthy controls. Similar postprandial alterations have been reported in individuals with type 2 diabetes mellitus [38]. Furthermore, several amino acid-derived analytes in the signature have also been implicated in metabolic dysfunction. Although postprandial profiles remain relatively underexplored in this context, serine and asparagine have shown differential responses to meal challenges in obesity-related studies [39–41]. One metabolite with particularly high discriminatory value, 2-hydroxybutyric acid, has been identified as a potential fasting-state biomarker for early insulin resistance and diabetes [40, 42].

The signature indicates that lipid metabolism was also disrupted in the presence of MetS, consistent with findings in the literature. Palmitic acid (C16:0) and heptadecanoic acid (C17:0) were among the lipids with the highest discriminatory value. Palmitic acid is well-known to be elevated in obesity and T2DM and is associated with insulin resistance and inflammatory signaling [42, 43]. While heptadecanoic acid is often considered a biomarker of dairy fat intake and has been inversely associated with T2DM risk in some studies, elevated levels may still reflect altered lipid handling in certain metabolic contexts. Stearic acid (C18:0) and palmitoleic acid (C16:1n7, also known as 9Z-hexadecenoic acid) showed smaller effect sizes. Stearic acid has been linked to altered fasting levels in obesity, whereas palmitoleic acid, a lipokine, has been reported to display altered postprandial dynamics in individuals with metabolic dysfunction [41, 43]. The sum of the free fatty acids has widely established connections to metabolic disease and obesity and has been shown to be differentially modulated postprandially [12, 44, 45].

Several analytes involved in carbohydrate metabolism, particularly glycolysis, appeared in the signature. Beyond glucose, which has been discussed earlier, D-mannose and 1,5-anhydroglucitol (1,5-AG) also showed altered postprandial profiles in MetS individuals. Both have been previously reported to exhibit altered fasting concentrations in metabolic disturbances [36]. However, we did not find prior studies characterizing their response dynamics in the context of a NCT. Pyruvic acid, a central glycolytic intermediate, has been implicated in the development of insulin resistance in skeletal muscle based on in vitro studies [46]. Although direct in vivo evidence is limited, its differential response here may support a broader metabolic inflexibility in MetS. Furthermore, γ-glutamyl transferase (GGT), a biomarker commonly associated with MetS, insulin resistance, and type 2 diabetes, also showed a significantly different postprandial response between groups. While its fasting associations are well documented, research on its acute response to NCTs is limited, making this finding a novel contribution to understanding GGT’s dynamic role in metabolic health.

The most enriched biological processes in the MetS biomarker signature were related to protein metabolism and lipoprotein metabolism (Fig. 3). In addition, pathways associated with core intermediary metabolism, lipolysis, and lipotoxicity featured prominently. This distribution is consistent with the well-established role of these processes in obesity-related metabolic disorders and underscores their centrality in the pathophysiology of MetS. At a systems level, many of the pathways enriched in the signature reflect liver-related metabolic functions, including lipid handling, glucose production, and amino acid processing. Adipose tissue function and elements of the systemic stress response (such as oxidative stress and inflammation) were also well represented, reflecting the multi-organ nature of metabolic dysregulation in MetS.

By assessing the overlap in analytes that were affected by the energy-restriction intervention, and analytes that were part of the MetS signature, we identified the subpanel of analytes that may be used as actionable dynamic postprandial biomarkers to monitor metabolic health during interventions. This subpanel primarily consisted of amino acids, including glutamate, leucine, glycine and tyrosine (Fig. 2). These amino acids have been associated with insulin resistance, prediabetes and increased risk of diabetes mellitus type 2 [47, 48]. In line with this observation, leucine and tyrosine have also been shown to respond to treatment with oral antidiabetic drug treatment [49]. Furthermore, leptin levels were significantly affected by the energy-restriction intervention and in fact was the analyte with the highest significance ratio (Fig. 3), indicating its potential application for monitoring treatment efficacy. At the biological process level, protein metabolism was the most prominently altered by the intervention, as expected based on amino acids found to be regulated by energy-restriction (Fig. 2). Additional changes were observed in processes related to core metabolism and beta-cell function, suggesting improvements in glucose regulation as a result of the energy-restriction intervention. Changes in lipid handling pathways also emerged, consistent with known effects of energy-restriction on lipolysis, fatty acid oxidation, and lipid transport [50]. At the tissue health level, muscle health appeared to be the second most affected domain, while adipose tissue health showed robust improvements, likely reflecting shifts in energy balance and fat mobilization. Alterations in the beta-cell function process further pointed to improved pancreatic health, underlining the multi-organ benefits of the energy-restriction.

A major strength lies in the multi-study data integration of four separate datasets, which increased the robustness and generalizability of the findings. Additionally, the methodological innovation in aggregating and visualizing multifaceted biological data provided a holistic view of the metabolic processes and physiological function domains impacted by MetS. This study further distinguishes itself by highlighting less-characterized analytes and their responses to NCTs, offering new leads for biomarker discovery. Although the identified signature comprises 64 analytes, it should be viewed as a discovery signature rather than a final clinical assay. Future studies should identify a smaller subset of biomarkers that retains predictive performance while improving feasibility for clinical implementation.

Several limitations should be acknowledged. Differences in analytical platforms, NCT composition and sampling protocols and study design may have introduced between-study variability, although recent work suggests that metabolic resilience readouts are relatively robust to variations in challenge test composition [51]. In addition, two studies had relatively small sample sizes, women were underrepresented, and the discovery analyses were cross-sectional in nature. Finally, dietary factors such as dairy intake may have contributed to the observed associations for heptadecanoic acid (C17:0). Future studies should validate these findings in larger, more balanced, and longitudinal cohorts.

## Conclusions

Our findings highlight the utility of dynamic biomarker responses to NCTs as sensitive indicators of metabolic health and intervention efficacy. By capturing key aspects of phenotypic flexibility, including biomarker trajectories and return to baseline, this approach provides a sensitive strategy to monitor metabolic resilience and support personalized lifestyle interventions.

## Supplementary Information

Below is the link to the electronic supplementary material.

Supplementary Material 1: Analytical platforms and measurements included in the multi-study analysis and independent NutriTech intervention study. The table provides an overview of the analytical platforms used in each study, the number of analytes measured per platform, and the corresponding measurement type (quantitative or semi-quantitative).

Supplementary Material 2: Characteristics of the 64 analytes identified by the multi-study analysis. For each analyte, the table reports the corresponding synonym, study coverage, statistical support across studies (number of significant ANOVA, fasting [T0] t-test, and AUC t-test results), fulfilment of the predefined effect size criterion, and assignment to one of the network-derived response clusters identified by community detection analysis.

## Data Availability

Access to Nutritech study data may be requested through https://dashin.eu/interventionstudies/. Data from studies described in Kardinaal et al.[16], Bakker et al. [15], van den Broek et al. [8] and Wopereis et al. [9] are available from the corresponding author upon reasonable request.

## Abbreviations

AUC: Area under the curve
BMI: Body mass index
ELISA: Enzyme-linked immunosorbent assay
FDR: False discovery rate
FFA: Free fatty acids
GGT: γ-glutamyl transferase
HDL: High-density lipoprotein
HDL-C: High-density lipoprotein cholesterol
HPLC: High-performance liquid chromatography
LDL: Low-density lipoprotein
LDL-C: Low-density lipoprotein cholesterol
MetS: Metabolic syndrome
m-knn: Mutual k-nearest neighbors
NCT: Nutritional challenge test
PhenFlex: Standardized mixed-meal nutritional challenge test
SD: Standard deviation
T2DM: Type 2 diabetes mellitus
TAG / TAGs: Triacylglycerides
1,5-AG: 1,5-Anhydroglucitol
