# Supplementary material for: Multi-study analysis reveals dynamic postprandial biomarkers for metabolic syndrome by low-level fusion of multi-platform data from nutritional challenge test studies
Source: Genes Nutr. 2026 Aug 17;21:27. doi: 10.1186/s12263-026-00817-9 (PMC13757235; doi:10.1186/s12263-026-00817-9)
Supplement: Supplementary file 1 [file 12263_2026_817_MOESM2_ESM.docx]

| **Study** | **Analytical platforms** | **Measurement type** |
| --- | --- | --- |
| Van den Broek et al. [8] | Clinical chemistry (n=12), ELISA (n=3) and GC-MS-metabolomics (n=26). | Quantitative and semi-quantitative |
| Wopereis et al. [9] | Clinical chemistry (n=14), MSD multiplex immunoassays (n=8), ELISA (n=3), and GC-MS-metabolomics (n=84). | Quantitative and semi-quantitative |
| Rundle et al. [14] | Clinical chemistry (n=10), ELISA (n=3), multiplex immunoassays (9), metabolic profiling LC-MS/MS (n=163), bile acids LC-MS/MS (n=13), GC-MS metabolomics (n=26) | Quantitative and semi-quantitative |
| Bakker et al. [15] | Clinical chemistry (n=16), multiplex kits (n=13), ELISA (n=4), LC-MS-lipidomics (n=108), LC-MS-fatty acids (n=21) and GC-MS-metabolomics (n=145). | Quantitative and semi-quantitative |
| Kardinaal et al. [16] | Clinical chemistry (n=9), ELISA (n=7), multiplex immunoassays (n=11) and oxylipin LC-MS-metabolomics (n=13). | Quantitative and semi-quantitative |
